# Nitric Oxide at the Nexus of ACE2 Biology and COVID-19: Implications for Cardiovascular and Neurodegenerative Comorbidities

**DOI:** 10.33549/physiolres.935729

**Published:** 2025-12-01

**Authors:** Olga PECHANOVA, Ludovit PAULIS

**Affiliations:** 1 Institute of Normal and Pathological Physiology, Centre of Experimental Medicine, Slovak Academy of Sciences, Bratislava, Slovak Republic; 2 Institute of Pathophysiology, Faculty of Medicine, Comenius University, Bratislava, Slovak Republic

**Keywords:** Nitric Oxide, ACE2, Angiotensin-(1-7), Mas Receptor, COVID-19, Endothelial Dysfunction, Hypertension, Obesity, Diabetes Mellitus, Neurodegeneration

## Abstract

SARS-CoV-2 engages ACE2 for cell entry, perturbing the counter-regulatory ACE2/Ang-(1–7)/Mas axis and shifting the renin-angiotensin system toward ACE/Ang II/AT1 signaling, with a concomitant reduction in nitric oxide (NO) bioavailability. NO sits at the crossroads of these pathways, acting both as an antiviral modulator of spike-ACE2 interactions and as a downstream mediator of Mas-dependent endothelial protection. This review summarizes evidence on NO across three layers: (i) viral entry (S-nitrosylation of spike/ACE2, protease modulation), (ii) cardiovascular comorbidities (hypertension, obesity, diabetes) where ACE2 downregulation impairs endothelial NO synthase (eNOS)-dependent NO production and promotes thrombosis and microvascular dysfunction, and (iii) neurovascular/neurodegenerative sequelae, in which renin-angiotensin-aldosterone system (RAAS) dysregulation along with imbalance between protective eNOS/nNOS and inflammatory iNOS fosters blood–brain barrier disruption, microthrombosis, and cognitive impairment. Shared mechanisms - endotheliitis, microvascular dysfunction, and neuroinflammation may explain convergent risks for cardiac injury and cognitive decline in long COVID-19. Putative therapeutic strategies may include restoring physiological NO (*via* Mas agonism, Ang-(1–7), inhibition of Ang 1–7 degradation and recombinant ACE2), pulmonary-selective inhaled NO, hybrid S-nitrosylated agents, and selective attenuation of iNOS/peroxynitrite alongside endothelial support. Targeted modulation - enhancing eNOS/nNOS while constraining iNOS offers a unified framework to mitigate both cardiovascular and neurodegenerative consequences of COVID-19.

## Entry of SARS-CoV-2 *via* ACE2 and the Protective ACE2/Ang-(1–7)/Mas Axis

SARS-CoV-2 uses angiotensin-converting enzyme 2 (ACE2) as its primary receptor for cell entry. The viral spike protein binds, *via* its receptor-binding domain (RBD), to the peptidase domain of ACE2. Subsequent proteolytic activation of spike protein by furin, transmembrane serine protease 2 (TMPRSS2), or other proteases enables fusion of the viral and cellular membranes or endosomal entry [1]. Regulation of ACE2 is multilayered: transcriptional and post-transcriptional mechanisms (e.g., microRNAs such as miR-421, miR-143, and the miR-200 family) substantially influence ACE2 expression [2] while post-translational modifications (glycosylation, phosphorylation) and cleavage by the metalloprotease a disintegrin and metalloproteinase 17 (ADAM17) generate a soluble form of ACE2 (sACE2) released into the circulation [3]. ACE2 therefore exists in both membrane-bound and soluble forms; the latter may act as a viral decoy by sequestering spike protein and limiting cell entry, a concept now tested in therapeutic studies employing recombinant human ACE2 (rhACE2, APN01) [3]. Upon SARS-CoV-2 binding, the ACE2 - virus complex is internalized, leading to functional down-regulation of ACE2 enzymatic activity [4].

Physiologically, ACE2 opposes the classical ACE/Ang II/AT1 axis. Its principal substrate is Ang II, which ACE2 hydrolyses to Ang-(1–7). ACE2 also converts Ang I to Ang-(1–9), later processed to Ang-(1–7) [5,6]. Ang-(1–7) acts *via* the Mas G-protein-coupled receptor to activate the phosphoinositide 3-Kinase (PI3K)/Akt pathway [7] and exerts vasodilatory, anti-proliferative, anti-fibrotic and anti-inflammatory (like inhibition of TGF-β, adhesion molecules and chemokine expression and NF-κB activity) actions [8,9]. Ang-(1–7)/Mas axis decreases platelet aggregation, promotes fibrinolysis, restrains NADPH oxidase activity, and activates antioxidant defenses such as the Nrf2/HO-1 pathway [10,11]. This ‘alternative renin-angiotensin system (RAS)’ is particularly engaged under hypoxic stress in heart, lung, and vascular beds, with multiple lines of evidence summarized in a recent focused review spanning myocardial infarction through COVID-19 [12].

**Fig. 1:**
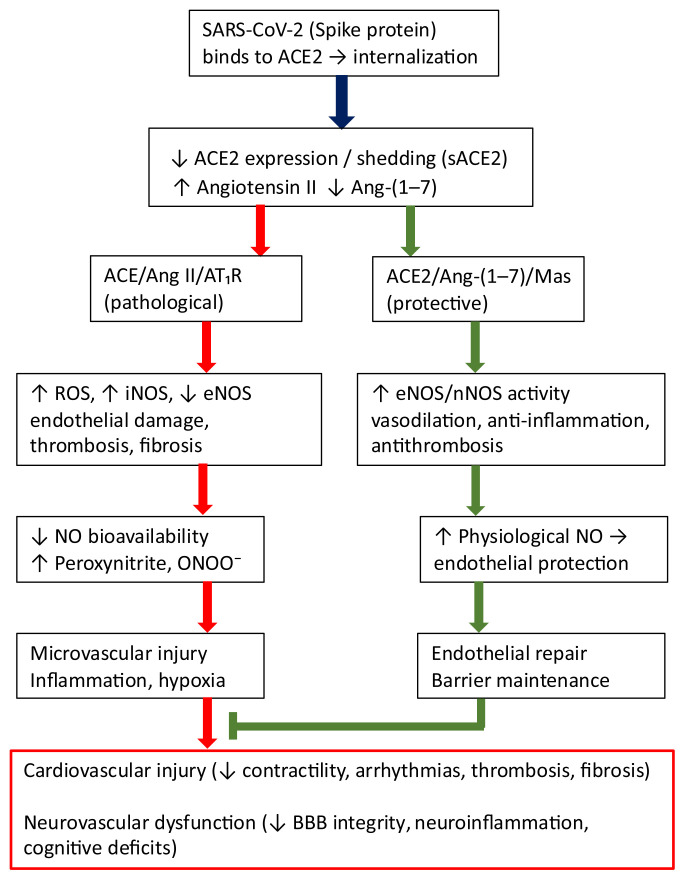
Nitric oxide (NO) at the crossroads of Angiotensin-Converting Enzyme2 (ACE2) biology in COVID-19. SARS-CoV-2 binding to ACE2 reduces ACE2/Ang-(1–7)/Mas signaling and shifts the renin–angiotensin balance toward ACE/Ang II/AT_1_R, leading to oxidative stress, endothelial nitric oxide synthase (eNOS) uncoupling, and excessive inducible nitric oxide synthase (iNOS)/peroxynitrite formation. The resulting endothelial dysfunction links systemic microvascular injury with cardiovascular and neurovascular sequelae. Restoration of physiological NO, e.g. through Mas activation, Ang-(1–7), recombinant ACE2, or selective iNOS inhibition may counteract these effects. Red arrows: Pathological pathways, Green arrows: Protective pathways

Clinical and experimental evidence indicates that loss of ACE2 exacerbates heart failure phenotypes, increases fibrosis, and compromises endothelial barrier integrity, whereas exogenous ACE2 or Ang-(1–7) confers cardioprotection [13] (Fig. 1). Beyond the cardiovascular system, the ACE2/Ang-(1–7)/Mas axis plays important roles in the kidney, lung, and brain, where it mitigates fibrosis, inflammation, and ischemic injury [14]. In the central nervous system (CNS), this pathway regulates blood pressure, microcirculatory flow and neuroinflammation through actions on astrocytes and microglia [6].

During SARS-CoV-2 infection, excessive internalization and downregulation of membrane ACE2 reduce conversion of Ang II to Ang-(1–7). The balance therefore shifts toward the ACE/Ang II/AT1 driven vasoconstriction, oxidative stress, inflammation and fibrosis [5]. This imbalance may also interact with the kallikrein–kinin system, as reduced ACE2 activity permits accumulation of des-Arg9-bradykinin and excessive B1/B2-receptor signaling, potentially worsening vascular leak and inflammation [15]. The shift is amplified in patients with hypertension, obesity, or cardiovascular disease, in whom the ACE2/Ang-(1–7)/Mas axis is often attenuated even before infection [5]. These insights support therapeutic strategies that activate the downstream ACE2/Ang-(1–7)/Mas axis, for example, recombinant ACE2, Ang-(1–7) administration, inhibition of Ang 1–7 degradation or Mas-receptor agonists to mitigate pulmonary, cardiovascular, and neurological complications of COVID-19 [6,16]

ACE2 thus serves as both the gateway for SARS-CoV-2 entry and the keystone of a protective counter-regulatory axis that culminates in nitric oxide (NO) release. Perturbations of ACE2 therefore inevitably translate into reduced NO bioavailability and endothelial injury.

## Role of NO in the Mechanism of SARS-CoV-2 entry and Protective ACE2/Ang-(1–7)/Mas Axis

Nitric oxide participates in the pathophysiology of COVID-19 not only as a regulator of vascular and immune functions but also by directly modulating viral entry into cells and the downstream effects of the protective ACE2/Ang-(1–7)/Mas axis. Studies have shown that NO can modify the interaction between the SARS-CoV-2 spike protein and its receptor ACE2 through S-nitrosylation of cysteine residues. This process alters protein conformation and reduces the virus ability to bind to target cells. In vitro experiments in cell lines (e.g., Vero E6) have confirmed that NO donors such as DETA-NONOate or S-nitrosoglutathione (GSNO) decrease the efficiency of viral binding to ACE2 and subsequent replication of SARS-CoV-2 [17]. NO can also S-nitrosylate viral cysteine proteases, including the main protease (M^pro), thereby impairing viral replication cycles in a mechanism analogous to that observed during the SARS-CoV-1 epidemic [18].

In addition, NO S-nitrosylates and inhibits cysteine proteases such as cathepsins, which participate in proteolytic processing of the spike protein. This processing is critical for fusion of the viral and cellular membranes, and its inhibition therefore directly restricts viral entry into host cells. A similar effect was described in SARS-CoV-1 epidemic, where NO inhibited viral replication by reducing spike palmitoylation and thereby diminishing its ability to interact with ACE2 [18]. Although these findings are largely based on pharmacological concentrations in vitro, they suggest that NO and its derivatives exert multi-level antiviral actions - on both viral and host proteins - a mechanism that may be presumed for SARS-CoV-2 as well.

NO is also a key downstream mediator of the protective ACE2/Ang-(1–7)/Mas axis. The binding of Ang-(1–7) to the Mas receptor activates the PI3K/Akt pathway leading to phosphorylation of endothelial NO synthase (eNOS) and increases NO generation [7]. Additional cross-talk with CaM- and AMPK-dependent pathways further enhances eNOS activity and vascular protection. NO then mediates the principal beneficial effects of this axis - inducing vasodilation, improving endothelial function, suppressing inflammation, and inhibiting proliferation of vascular smooth muscle cells.

When ACE2 is downregulated, for example after SARS-CoV-2 infection, activity of the Ang-(1–7)/Mas axis and eNOS signaling decline, leading to reduced NO levels. Importantly, oxidative stress associated with Ang II/AT1 overactivation rapidly scavenges NO *via* superoxide, limiting its bioavailability even when synthesis is partially preserved. NO reduction is one of the main reasons why loss of ACE2 results in endothelial dysfunction, thrombosis, and vascular complications [19]. In the cardiovascular system, NO mediates the vasorelaxant and antifibrotic actions of Ang-(1–7) and helps prevent remodeling of the heart and vasculature [6]. In the brain, this pathway contributes to regulation of cerebral blood flow, maintenance of blood–brain barrier integrity, and neuroprotective processes [14].

Thus, in the context of COVID-19, NO has a dual role. On the one hand, it acts as a direct antiviral and host-protective factor that disrupts the interaction of SARS-CoV-2 with ACE2 and inhibits viral replication. On the other hand, it serves as a downstream effector of the ACE2/Ang-(1–7)/Mas protective axis, conveying its vasodilatory, antithrombotic, and anti-inflammatory effects. This combination of actions helps explain why NO donors (e.g., inhaled NO or S-nitrosothiols) are being investigated as potential therapeutic strategies for both the acute phase of COVID-19 and its long-term sequelae. The intertwined mechanisms also link viral entry to systemic endothelial dysfunction, which becomes most evident in patients with cardiovascular comorbidities.

## ACE2 Downregulation in Cardiovascular Comorbidities of COVID-19

Disruptions in NO signaling are critically implicated in cardiovascular complications. These represent the major risk factors for severe disease and provide the pathophysiological background in which SARS-CoV-2-induced ACE2 downregulation exerts the greatest harm [3].

Patients with hypertension belong to the highest-risk groups in COVID-19. Elevated blood pressure is associated with increased activity of the ACE/Ang II/AT1 axis and, frequently, reduced ACE2 expression [5]. Experimental data indicate that chronic Ang II elevation suppresses ACE2 mRNA *via* AT1-dependent signaling, whereas ACE inhibition or AT1 blockade restore ACE2 expression. Following SARS-CoV-2 infection, this deficit is further exacerbated. Clinical studies have reported hypertension in 30–50% of hospitalized COVID-19 patients, significantly increasing mortality risk [19,20]. Mechanistically, the hypertensive phenotype is characterized by augmented endothelial dysfunction, arterial stiffness, and a pro-thrombotic tendency when ACE2 function is decreased [21]. Key endothelial biomarkers, such as endothelin-1, von Willebrand factor, and soluble adhesion molecules are elevated in hypertensive COVID-19 patients, reflecting systemic endothelial damage [22]. Experimental studies in spontaneously hypertensive rats (SHR) demonstrate that lowering ACE2 worsens cardiac dysfunction and promotes myocardial fibrosis [23]. Importantly, treatment with ACE inhibitors or AT1-receptor blockers has not been shown to increase infection risk and remains recommended by professional societies during COVID-19 [24].

Obesity is another major risk factor. Adipose tissue expresses ACE2 while also producing pro-inflammatory cytokines (IL-6, TNF-α) that contribute to a cytokine storm. Obesity is commonly accompanied by insulin resistance, dyslipidemia, and chronic low-grade inflammation, leading to dysregulation of the RAS and elevated Ang II levels [25]. Retrospective analyses consistently show obesity as an independent predictor of hospitalization, mechanical ventilation, and mortality in COVID-19 [26]. In a cohort study, obesity was identified as an independent predictor of severe disease in patients younger than 60 years [27]. Adipose inflammation and endothelial dysfunction together create a pro-thrombotic milieu that intensifies when ACE2 activity is reduced.

In patients with diabetes mellitus (particularly type 2), ACE2 function is likewise impaired. Chronic hyperglycemia augments ACE/Ang II/AT1 signaling and fosters oxidative stress, leading to endothelial dysfunction and microangiopathy [28]. Clinical data indicate that diabetes was present in 20–30% of hospitalized COVID-19 patients and was associated with greater severity and mortality [29,30]. Pathophysiologically, “diabetic cardiomyopathy” characterized by oxidative stress, fibrosis, and metabolic derangements is exacerbated by decrease of ACE2 protective activity [31]. Experimental models confirm that ACE2-deficient mice exhibit increased susceptibility to diabetic nephropathy and cardiomyopathy [32]. Insulin resistance additionally reduces PI3K/Akt-eNOS signaling, further limiting endothelial NO bioavailability.

The combination of hypertension, obesity, and diabetes - common in the metabolic syndrome creates a syndemic effect in COVID-19. Clinically, this manifests as a higher risk of acute myocardial injury (troponin elevation in up to 20–30% of critically ill patients) [33], more frequent arrhythmias, acute heart failure, pulmonary embolism, and disseminated intravascular coagulation [34]. Autopsy studies in COVID-19 have shown diffuse alveolar damage accompanied by capillary congestion and microthrombi in the lungs and myocardium [35].

ACE2 expression and NO signaling decline with aging and are lower in males, which may partially explain the higher mortality observed in older men [36]. These findings highlight that pre-existing attenuation of the ACE2/Ang-(1–7)/Mas/eNOS axis predisposes to severe endothelial injury when additional downregulation occurs during SARS-CoV-2 infection.

## Role of NO in ACE2 Downregulation and Cardiovascular Comorbidities of COVID-19

When ACE2 is downregulated, the Ang-(1–7)/Mas/eNOS pathway is suppressed and bioactive NO declines. Concurrently, overactivation of the Ang II/AT1 axis increases the generation of reactive oxygen species (ROS) *via* NADPH oxidases, which rapidly inactivate NO and drive oxidative stress [21]. Finally, the augmentation in the classical RAAS signaling in COVID-19 aligns with changes in aminopeptidase A and M activities (APA/APM → Ang III/IV) and bradykinin dysregulation linking it to endothelial dysfunction [37]. The result is marked endothelial dysfunction, impaired vasodilation, enhanced platelet adhesion and aggregation, and pro-inflammatory endothelial activation [38].

In addition to a deficit of physiological eNOS-derived NO, severe inflammation and the “cytokine storm” induce inducible nitric oxide synthase (iNOS) in immune and vascular cells, leading to sustained, high-output NO production. In an oxidant-rich milieu, NO rapidly combines with superoxide to form peroxynitrite (ONOO^−^), which nitrates proteins (3-nitrotyrosine) and oxidizes lipids, damaging endothelium and myocardium and contributing to vasoplegia, impaired vasoreactivity, and microvascular injury. Increased iNOS activity and/or elevated iNOS levels have been reported in severe COVID-19, correlating with ventilatory failure and cardiac injury, which illustrates NO and iNOS as a biological “double-edged sword” [7,21].

These mechanisms amplify cardiovascular risk in patients with hypertension, obesity, and diabetes. In hypertension, oxidative stress and elevated Ang II further uncouple eNOS and reduce NO even before infection; SARS-CoV-2 accelerates this process, worsening vascular stiffness and thrombosis [39]. In obesity and diabetes, chronic oxidative and inflammatory stress associated with insulin resistance and eNOS dysfunction means that decrease of ACE2 and reduced NO further promote atherothrombosis and myocardial fibrosis [40–42]. Clinically, this NO deficit manifests as higher rates of microthrombosis, pulmonary embolism, and acute myocardial injury in patients with severe COVID-19 [35].

Taken together, in the periphery, ACE2 downregulation unbalances NO homeostasis on two fronts: (i) loss of protective eNOS-derived NO due to suppressed Ang-(1–7)/Mas signaling and oxidative inactivation, and (ii) pathological iNOS induction with peroxynitrite overproduction. These changes, however, affect the central nervous system as well.

## Neurovascular and Neurodegenerative Consequences of ACE2 Downregulation

ACE2 is expressed not only in the cardiovascular and pulmonary systems but also in the brain - localised to neurons, astrocytes, oligodendrocytes, and the cerebrovascular endothelium [4]. Although overall brain ACE2 expression is relatively low, it is abundant in endothelium, pericytes and astrocytes, supporting a predominantly vascular/perivascular role of the ACE2/Ang-(1–7)/Mas axis in the CNS. Nevertheless, besides the ACE2/Ang-(1–7)/Mas axis playing a pivotal role in regulating cerebral blood flow, it modulates neurovascular coupling, inflammatory signaling, and blood–brain barrier integrity as well. Consequently, ACE2 downregulation during SARS-CoV-2 infection affects not only respiration and the cardiovascular system but also has substantial neurological repercussions.

ACE2 downregulation promotes accumulation of Ang II and hyperactivation of AT1 receptors, leading to vasoconstriction, increased blood–brain barrier permeability, and endothelial dysfunction. In experimental models of ischemic stroke, ACE2-deficient mice exhibit larger infarcts, greater neuronal apoptosis, and more pronounced blood–brain barrier damage [14]. Conversely, administration of Ang-(1–7) or activation of the Mas receptor improves microcirculation and reduces inflammation and fibrosis [43]. Clinically, neurological complications of COVID-19 (encephalopathy, acute ischemic stroke, intracranial hemorrhage) have been linked to endotheliitis, microthrombosis, and impaired cerebral autoregulation [44].

With ACE2 downregulation, expression of pro-inflammatory cytokines (IL-6, TNF-α, IL-1β) and NF-κB activity increases, driving astrocyte and microglial activation [45]. This state fosters neuroinflammation, which is associated with synaptic injury and progression of neurodegenerative disorders. Increasing Ang II/AT1 signaling further stimulates NADPH oxidases (NOX2, NOX4) and ROS generation, intensifying oxidative stress and mitochondrial dysfunction in neurons [46]. In parallel, reduced Ang-(1–7)/Mas–eNOS signaling lowers NO bioavailability at the neurovascular unit, weakening vasodilatory reserve and blood–brain barrier integrity and exacerbating hypoperfusion-injury cascades.

Disruption of the ACE2/Ang-(1–7)/Mas axis is linked to multiple neurodegenerative processes. In Alzheimer disease, reduced ACE2 levels have been detected in brain tissue and cerebrospinal fluid [47]. ACE2 reduction promotes amyloidogenesis and tau hyperphosphorylation *via* oxidative and inflammatory pathways. In Parkinson disease, increased Ang II/AT1 activity in the substantia nigra contributes to neuroinflammation and dopaminergic neuron reduction [48]. By contrast, Mas-receptor activation exerts neuroprotective effects. In ischaemic stroke, experimental and clinical data indicate that ACE2 protects the brain by lowering oxidative stress, improving perfusion, and suppressing excitotoxicity [4,35,49]. Beyond these associations, accumulating evidence suggests that RAAS-NO imbalance intersects with aberrant S-nitrosylation of neuronal proteins, a mechanism implicated in synaptic dysfunction across neurodegenerative disorders [50].

Neurological complications are really common in COVID-19. Meta-analyses indicate that approximately 30–40% of hospitalized patients present with neurological symptoms, including headache, anosmia, encephalopathy, stroke, or seizures. [51]. Risk is further increased in those with comorbid hypertension, diabetes, or obesity, consistent with pre-existing dysregulation of ACE2 and the RAS in the brain [52]. Hypertension and endothelial dysfunction worsen cerebral autoregulation and contribute to chronic cerebral hypoperfusion. In long COVID, concomitant endotheliitis and microthrombi are hypothesized to accelerate vascular dementia and cognitive impairment [53]. Obesity, characterized by low-grade systemic inflammation and insulin resistance, promotes neuroinflammation. In long COVID, obesity has emerged as a predictor of more severe and persistent cognitive symptoms [54]. Diabetes augments protein glycation, oxidative stress, and endothelial dysfunction. When combined with post-COVID ACE2 downregulation, this accelerates neurovascular injury and increases the risk of Alzheimer disease and vascular dementia [55].

Long COVID is associated with persistent cognitive dysfunction, attentional deficits, and memory impairment, likely driven by chronic dysregulation of the ACE2/Ang-(1–7)/Mas axis and sustained neuroinflammation. Large population-based studies show increased risks of cognitive and neurological diagnoses months after the acute phase: in a retrospective cohort of >230,000 patients, 33% had a neurological or psychiatric diagnosis within 6 months of infection [56]. Patients with comorbid hypertension, obesity, or diabetes exhibited higher rates of cerebrovascular events, memory disorders, and dementia [57], and other studies report increased prevalence of “brain fog,” attentional deficits, and executive dysfunction particularly in patients with diabetes and obesity following COVID-19 [58].

In summary, ACE2 downregulation during SARS-CoV-2 infection destabilizes the neurovascular unit, increases blood–brain barrier permeability, and drives neuroinflammation and oxidative stress. These changes might accelerate neurodegenerative processes and elevate the risk of both acute neurological events and chronic cognitive impairment. Restoring or enhancing ACE2/Ang-(1–7)/Mas signaling and/or preserving NO bioavailability at the neurovascular unit constitutes a promising neuroprotective strategy in COVID-19 and in neurodegenerative disease contexts.

## Role of NO in the Neurodegenerative Context of COVID-19

The balance between protective eNOS/nNOS signaling and deleterious iNOS activity is critical in both, the cardiovascular system and the brain. Downregulation of ACE2 and the ensuing dysregulation of NO therefore translate not only into cardiovascular complications but also into neurovascular and neurodegenerative consequences of COVID-19. Clinically, a deficit of bioactive NO manifests as vasoconstriction, enhanced platelet aggregation, and a propensity for microthrombosis. These mechanisms are supported by autopsy studies in COVID-19 demonstrating diffuse endotheliitis and capillary microthrombi [35].

Conversely, increased iNOS activity during hyperinflammation can lead to NO overproduction and formation of peroxynitrite, which injures endothelium and myocardium *via* nitro-oxidative stress [59]. This creates a paradoxical scenario: a lack of protective NO in the microcirculation is accompanied by harmful overproduction of reactive NO derivatives.

In the nervous system, NO likewise plays a dual role. Under physiological conditions, it improves cerebral perfusion, maintains blood–brain barrier integrity, and contributes to synaptic plasticity and memory processes [60]. In SARS-CoV-2 infection, however, ACE2 dysregulation diminishes the protective eNOS/nNOS component while increasing iNOS activity as a consequence of neuroinflammation. This milieu promotes impaired neurovascular coupling and cerebral hypoperfusion, increased blood–brain barrier permeability, formation of peroxynitrite and nitrotyrosine that damage neurons and mitochondria, and dysregulated S-nitrosylation of key proteins (e.g., Drp1, Parkin, NMDA receptors), culminating in synaptic dysfunction and apoptosis [61].

These processes are shared across the pathogenesis of neurodegenerative disorders such as Alzheimer and Parkinson diseases and may help explain why patients with long COVID have an elevated risk of cognitive impairment [62]. In the CNS, NO is a central player in the pathophysiology of COVID-19 - its endothelial deficit contributes to vascular complications, whereas iNOS-driven overproduction under inflammatory conditions leads to neurotoxic sequelae. The balance between protective and harmful NO activity may determine disease course and the emergence of long-term complications, including neurodegeneration. Therapeutic strategies aimed at restoring physiological NO signaling (e.g., preserving eNOS coupling/BH_4_ sufficiency and limiting iNOS-driven peroxynitrite) represent a promising way to mitigate the neurological consequences of COVID-19.

## Nitric Oxide–Based Therapeutic Strategies in the Context of COVID-19 Comorbidities

The duality in NO function implies that therapy should neither broadly stimulate nor globally inhibit NO, but rather selectively modulate its sources, local production, and downstream actions (Table 1).

One approach is to restore physiological eNOS signaling, which is tightly linked to the protective ACE2/Ang-(1–7)/Mas axis. Binding of Ang-(1–7) to the Mas receptor activates the PI3K/Akt pathway and phosphorylates eNOS, thereby augmenting bioactive NO production [7]. Experimental studies demonstrate that stimulating this pathway improves endothelial function, reduces inflammation and vascular remodeling, and confers both cardioprotective and neuroprotective benefits [6]. Another experimental approach might include dual RAS inhibition blocking Ang II formation and reducing Ang 1–7 degradation simultaneously, such as being investigated for pulmonary hypertension [16]. Clinically, Ang-(1–7) analogues, Mas-receptor agonists, and recombinant ACE2 are being explored as candidate therapeutics. Here the early clinical testing of Mas-axis augmentation in severe COVID-19 has shown acceptable safety but no consistent efficacy on hard outcomes, suggesting that patient selection and timing may be critical [63], while recombinant ACE2 remains mechanistically attractive but still requires confirmation of clinical benefit beyond biochemical target engagement.

A second strategy are NO donors and inhaled NO [64–67]. Inhaled NO has been used for more than two decades in acute respiratory distress syndrome (ARDS) and improves oxygenation and lowers pulmonary hypertension with minimal systemic effects [65]. In COVID-19, several clinical studies suggest improved oxygenation parameters and reduced viral replication with inhaled NO in severe disease, yet without a consistent survival benefit [66,67].

S-nitrosylated drug derivatives, such as S-nitrosocaptopril, merit particular attention. These hybrid molecules combine ACE inhibition with controlled NO donation, potentially replenishing physiological NO while dampening detrimental Ang II/AT1 signaling. Experimental work indicates that S-nitrosocaptopril releases captopril (and its disulfide metabolite) alongside NO and NO_2_, yielding a distinctive cardioprotective and potentially neuroprotective profile [68]. On top of this mechanism, targeted S-nitrosylation strategies that modify host/viral proteins—most notably ACE2—have shown preclinical inhibition of spike-ACE2 binding and viral entry, but clinical data are not yet available [69].

Conversely, during hyperinflammation it is desirable to selectively attenuate iNOS-derived NO or scavenge its toxic products, notably peroxynitrite. Selective iNOS inhibitors and antioxidants capable of neutralizing reactive nitrogen species (e.g., tempol, ebselen, urate) are under investigation [70–72].

Also, supporting endothelial function and indirectly increasing eNOS bioactivity with widely used agents remains important. Statins, ACE inhibitors, and AT1-receptor blockers (ARBs) improve endothelial function and enhance NO bioavailability [73–75]. Supplementation with NOS substrates such as L-arginine is being evaluated in clinical studies of post-COVID syndrome characterized by endothelial dysfunction [76]. Small randomized studies of L-arginine (± vitamin C) improved flow-mediated dilation, 6-minute walk distance, and fatigue in long COVID, and reduced need for respiratory support/length of stay in hospitalized patients [77,78], but further confirmation in larger, multicenter RCTs is warranted.

Finally, appropriate diet and nutrition, providing relevant amounts of nitrate and antioxidant compounds such as polyphenols, may support the restoration of physiological NO levels in long-COVID, *via* the enterosalivary nitrate–nitrite–NO pathway and by limiting oxidative scavenging of NO and preserving eNOS coupling. However, current clinical evidence remains limited and largely derived from small trials [79–81].

Taken together, these observations argue for a differentiated NO-centered therapeutic strategy in COVID-19 - strengthen physiological eNOS/nNOS signaling, curb pathological iNOS overproduction, and maintain a balance between protective and deleterious NO effects. Looking ahead, hybrid approaches that combine RAS modulation with controlled NO release are emerging as particularly promising.

## Conclusion

ACE2 downregulation following SARS-CoV-2 infection disrupts the ACE2/Ang-(1–7)/Mas axis and reduces NO bioavailability, while inflammation-induced iNOS generates toxic NO derivatives. Together with eNOS uncoupling under oxidative stress, this dual hit to NO homeostasis links endothelial dysfunction, microthrombosis, and neurovascular dysfunction across organs. Evidence supports therapeutic approaches that (i) restore physiological NO production *via* Mas/eNOS signaling, (ii) selectively suppress iNOS/peroxynitrite, and (iii) combine RAS modulation with controlled NO release.

Future studies should stratify patients by comorbidities (hypertension/obesity/diabetes mellitus), standardize biomarkers of endothelial and cognitive dysfunction, and test combination regimens targeting shared microvascular mechanisms. These strategies should be assessed in adaptive, randomized trials incorporating mechanistic endpoints such as plasma nitrite/nitrate, 3-nitrotyrosine, flow-mediated dilation, and cerebrovascular reactivity alongside clinically meaningful outcomes (thrombotic events, cardiac injury, and cognitive performance). Careful attention to timing (acute vs convalescent/long-COVID phases), route and dose to favor eNOS/nNOS while avoiding iNOS-driven nitrosative stress will be pivotal for safety and efficacy.

## Figures and Tables

**Table 1:** Therapeutic strategies targeting the ACE2–NO axis in COVID-19 and post-COVID complications

Therapeutic strategy	Primary molecular target / mechanism	Expected effect on NO pathway	Level of evidence	Key limitations / comments	Refs.
**Recombinant human ACE2 (rhACE2)**	Decoy receptor for SARS-CoV-2; restores ACE2/Ang-(1–7)/Mas balance	↑ Ang-(1–7), ↑ eNOS/nNOS, ↑ physiological NO	*Preclinical, Early Phase I–II*	Short plasma half-life; limited clinical data; ongoing phase II trials	[3]
**Ang-(1–7) peptide / Mas receptor agonists**	Activation of Mas receptor and PI3K/Akt pathway; counteracts ACE/Ang II/AT_1_R axis	↑ eNOS/nNOS activation, ↓ oxidative stress, ↓ iNOS	*Preclinical + Small animal models*	Limited stability of native peptide; need for oral/long-acting analogues	[6, 7, 63]
**NO donors (e. g. nitroglycerin, sodium nitroprusside, SNAP, S-nitrosothiols)**	Direct NO release / S-nitrosylation	↑ NO bioavailability; vasodilation; antiviral S-nitrosylation of viral proteins	*Preclinical + limited clinical*	Systemic hypotension; short duration; non-selective action	[64]
**Inhaled nitric oxide (iNO)**	Pulmonary-selective vasodilator; antiviral and anti-thrombotic	Local ↑ NO, improved V/Q matching	*Phase II–III (mixed results)*	No proven survival benefit; transient improvement in oxygenation	[65–67]
**L-arginine / L-citrulline supplementation**	Substrate supply for NOS enzymes	↑ eNOS/nNOS-derived NO	*Small RCTs (supportive evidence)*	Small cohorts; metabolic variability; possible pro-oxidant shift if BH_4_ deficient	[76–78]
**iNOS inhibitors (e.g. 1400W, aminoguanidine)**	Selective inhibition of inducible NOS	↓ pathologic NO/peroxynitrite formation	*Preclinical only*	Safety, off-target effects, lack of human data	[70, 71]
**S-nitrosocaptopril and hybrid NO-donor ACE inhibitors**	ACE inhibition + controlled NO release	↓ Ang II, ↑ NO bioavailability	*Preclinical*	No clinical data in COVID-19; stability and dosage yet to be optimized	[68, 69]
**Statins, ACE inhibitors, ARBs (repurposed)**	Indirect restoration of endothelial ACE2/NO coupling	↑ eNOS activity, ↓ oxidative stress	*Large retrospective + some RCTs*	Heterogeneous outcomes; need mechanistic trials	[73–75]
**Dietary nitrate/nitrite supplementation (beetroot juice, NaNO** **_2_** **)**	eNOS-independent nitrate–nitrite–NO pathway	↑ plasma nitrite/NO, improved endothelial function	*Preclinical + pilot human studies*	Variable bioavailability; short-term benefit only	[79]
**Antioxidants (vitamin C, polyphenols N-acetylcysteine)**	ROS scavenging, preservation of NO	↑ bioavailability indirectly	*Variable preclinical + small trials*	Mixed results; no clear mortality benefit	[80, 81]

ACE2 – Angiotensin-Converting Enzyme 2; rhACE2 – Recombinant human ACE2; Ang-(1–7) – Angiotensin-(1–7); Mas – Mas receptor; ACE – Angiotensin-Converting Enzyme; Ang II – Angiotensin II; AT_1_R – Angiotensin II type 1 receptor; eNOS – Endothelial Nitric Oxide Synthase; nNOS – Neuronal Nitric Oxide Synthase; iNOS – Inducible Nitric Oxide Synthase; NO – Nitric Oxide; PI3K – Phosphoinositide 3-Kinase; Akt – Protein kinase B; SARS-CoV-2 – Severe Acute Respiratory Syndrome Coronavirus 2; V/Q matching – Ventilation/Perfusion matching; SNAP – S-nitroso-N-acetylpenicillamine; S-nitrosothiols – S-nitrosylated thiol derivatives; RCT – Randomized Controlled Trial; BH_4_ – Tetrahydrobiopterin; ROS – Reactive Oxygen Species; NaNO_2_ – Sodium nitrite; ARBs – Angiotensin II Receptor Blockers.

## Abbreviations

ACE: Angiotensin-Converting Enzyme
ACE2: Angiotensin-Converting Enzyme 2
ADAM17: A Disintegrin and Metalloproteinase 17
AKT: Protein Kinase B
AMPK: AMP-Activated Protein Kinase
Ang I: Angiotensin I
Ang II: Angiotensin II
Ang-(1,7): Angiotensin-(1,7)
Ang-(1,9): Angiotensin-(1,9)
APA: Aminopeptidase A
APM: Aminopeptidase M
ARBs: Angiotensin II Receptor Blockers
AT_1_/AT_1_R: Angiotensin II Type 1 Receptor
BH_4_: Tetrahydrobiopterin
CaM: Calmodulin
CNS: Central Nervous System
COVID-19: Coronavirus Disease 2019
DETA-NONOate: Diethylenetriamine NONOate (NO donor)
Drp1: Dynamin-related protein 1
eNOS: Endothelial Nitric Oxide Synthase
GSNO: S-Nitrosoglutathione
HO-1: Heme Oxygenase 1
IL-1β: Interleukin 1 beta
IL-6: Interleukin 6
iNOS: Inducible Nitric Oxide Synthase
Mas: Mas Receptor (receptor for Ang-(1,7))
M^pro: Main Protease (3C-like protease) of SARS-CoV-2
miR: microRNA
NF-κB: Nuclear Factor kappa B
nNOS: Neuronal Nitric Oxide Synthase
NO: Nitric Oxide
NO_2_: Nitrogen Dioxide
NOX2: NOX4, NADPH Oxidase Isoforms 2 a 4
Nrf2: Nuclear Factor Erythroid 2, Related Factor 2
ONOO^−^: Peroxynitrite
PI3K: Phosphoinositide 3-Kinase
RAS: Renin, Angiotensin System
RAAS: Renin, Angiotensin, Aldosterone System
RCT: Randomized Controlled Trial
RBD: Receptor-Binding Domain (spike protein)
rhACE2: Recombinant Human Angiotensin-Converting Enzyme 2
ROS: Reactive Oxygen Species
SARS-CoV-1/SARS-CoV-2: Severe Acute Respiratory Syndrome Coronavirus 1/2
sACE2: Soluble ACE2
SHR: Spontaneously Hypertensive Rat
SNAP: S-Nitroso-N-Acetylpenicillamine (NO donor)
TACE: TNF-α,Converting Enzyme (ADAM17)
TGF-β: Transforming Growth Factor Beta
TMPRSS2: Transmembrane Serine Protease 2
TNF-α: Tumor Necrosis Factor Alpha
V/Q matching: Ventilation/Perfusion Matching
WHO: World Health Organization
